# Strengthening TB surveillance system in India: Way forward for improving estimates of TB incidence

**DOI:** 10.4103/0970-2113.80325

**Published:** 2011

**Authors:** Rinku Sharma, Vivek Jain, Saudan Singh

**Affiliations:** *Department of Community Medicine, Vardhman Mahavir Medical College and Safdarjung Hospital, New Delhi, India*

**Keywords:** Indicator, Styblo’s rule, surveillance, tuberculosis

## Abstract

One of the key indicators used under India’s TB control program is the DOTS case detection rate of new sputum smear positive TB whose estimates in India are based on incidence estimates derived from Styblo’s rule. Styblo’s rule was formulated in an era without well-established tuberculosis control program, effective tuberculosis drugs, and emergence of TB-HIV coinfection, so today it does not reflect the true incidence of TB. Considering various loopholes in different methods of measurement of incidence (prevalence surveys of disease/ infection, vital registration system and Styblo’s rule), strengthening of existing surveillance system is the best tool to obtain correct estimates of tuberculosis incidence in India.

## INTRODUCTION

Tuberculosis control has been a long-term battle since the discovery of tuberculosis bacilli, by Robert Koch 125 years back. Global tuberculosis control efforts have made major progress in the past decade. One such effort is part of the millennium development goals (MDGs), which are unique in themselves, as a commitment by 189 countries to meet the need of the world’s poorest. Under MDGs, goal 6 specifies to combat HIV/AIDS, malaria and other diseases. In Indian scenario, among other diseases tuberculosis is of particular importance because of its relation to high morbidity and mortality.[1] In relation to tuberculosis, target 8 specifies “to stop increase and reverse the incidence of tuberculosis.” These goals and targets have been translated into certain indicators to assess the progress of different countries toward their achievement. For tuberculosis, indicator 23 is “to half the prevalence and mortality due to tuberculosis as per 1990 estimates” and indicator 24 is “to achieve cure rate of 85% among new sputum smear positive cases and case detection rate of 70% among the same.” To be able to assess our progress toward MDGs, we ought to have correct estimates of incidence, prevalence, and deaths due to tuberculosis.

### Estimating tuberculosis burden

At present TB incidence, prevalence, and mortality in a particular year can be measured by five different methods:

#### Indirect estimation of TB incidence using surveys of the annual risk of TB infection

In India till now series of surveys of the annual risk of infection have been the only method used to measure TB incidence and was also used to produce WHO estimates, as up till mid-2008. These estimates are based on the *Styblo rule* which was formulated in an era without established tuberculosis control program, efficacious treatment and before the emergence of HIV epidemic. It was based on few assumptions:[2]

- Each prevalent smear positive case makes about 10 contacts per year (*b* = 10).
- Case fatality rate among untreated cases is 50% and untreated cases remains smear positive for an average of 2 years.
- Incidence of the infection/disease was estimated by dividing the prevalence of the infection or disease by 2 (*P* = 2I) using the standard formula, *P* = ID.
- Conventionally, it was also thought that the lifetime risk of progressing from infection to active tuberculosis is about 10%.[3]

Annual risk of tuberculosis infection (ARTI) in a particular population depends on prevalence of infection (*P*) and number of infectious contacts per smear positive case (*b*), then ARTI = *bP*, or 10 × 2 × I. Incidence of new smear positive cases per 100 000 population per percent increase in ARTI would work out to [I × (100 000/ARTI) × 100] = I × 100 000/10 × 2 × *I* × 100= 50, it implies that an increase in ARTI of 1% per year corresponds with an increase of 50 smear positive cases per 100 000 population per year.

The major obstacles in deriving the annual risk of tuberculosis infection are related to factors such as assumption of no change in prevalence of infection over the calendar year; operational difficulty in obtaining adequate sample of children; varying and unpredictable specificity of tuberculin skin test as a result of nonspecific sensitization to environmental mycobacteria as well as type and extent of BCG vaccination; reduced sensitivity of tuberculin skin test due to growing impact of HIV; and issues related to different techniques to estimate the proportion of reactors to tuberculin skin testing, e.g., uncertainties around cut off points despite complicated analysis such as mirror image and mixture analysis, observed digit preference while measuring tuberculin reactions, etc.

#### Direct measurement of TB incidence from TB notification data

It is the most preferred method for measuring the TB incidence. This method is only valid if there is strong evidence that all TB cases are diagnosed and notified.

#### Direct measurement of TB incidence

TB incidence is measured either by a prospective follow-up of a population cohort, or by doing two consecutive prevalence surveys within a short time span and estimating the number of new cases occurring in between the surveys (TB cases that are picked up by the second survey and not during the first survey). In practice, carrying out longitudinal cohort studies are not possible, because it requires several hundred thousand of individuals who need to be followed up, which may not be feasible in a developing country like India.

#### Indirect estimation of TB incidence using TB disease prevalence surveys

Tuberculosis prevalence surveys are most valuable in areas where notification data obtained through routine surveillance are of unproven accuracy or incomplete. Deriving incidence from prevalence surveys requires a good estimate of the duration of disease, which is difficult to obtain from prevalence surveys. Logistically, conducting prevalence survey is also quite a challenging job. A survey of the prevalence of TB disease will not identify cases of TB that are not confirmed by smear or culture (particularly HIV positive cases). Despite having all these limitations periodic assessment of prevalence surveys are useful for measuring the short term impact of TB control (such as within five years) as in the current situation, when both improved case finding and effective treatment under RNTCP shorten the duration of disease, as prevalence responds more rapidly than incidence to changes in TB control.

#### Indirect estimation using mortality data recorded in vital registration systems

TB incidence is estimated as the number of TB deaths divided by the estimated case-fatality rate. There are three ways to estimate the mortality in India. First is through vital registration system which is currently unreliable due to under registration of deaths. To cite an example, only one third of the estimated annual deaths were registered in India during 1999.[4] Moreover, among the registered deaths, the cause of death were available for one in three deaths and this system often lumps together the deaths as due to accidents, violence, and disease without further details. The second system to estimate the mortality is through medically certified cause of death. However, it covers only a small population and is largely confined to urban settings. Moreover, it suffers from inconsistent physician attribution of cause of death. The third method is sample registration system (SRS), which operates through its randomly selected units in rural and urban area in India. Major drawback of SRS is that it cannot provide district level data and it also lacks sufficient power to generate yearly rates for less common causes of deaths. In India where the vital registration system is weak, sample vital registration with validated verbal autopsy is the most promising interim solution for reliably measuring deaths (including deaths from TB)[5] This system will be helpful in estimating case fatality rate outside DOTS programs, which are much harder to measure.

### Estimating the True burden of TB in India

There cannot be a good alternative other than the development of a comprehensive and effective surveillance system to have correct estimates of tuberculosis burden (mentioned as method 1 in the above text). According to WHO a good surveillance system must focus on last four components of Stop TB strategy 2006.[6] Steps taken in India to improve the TB surveillance system based on WHO Stop TB strategy are:

#### Health system strengthening through

- Integration of tuberculosis control activities with primary health care services: In rural area RNTCP, as being an integral part of the NRHM (National Rural Health Mission) is implemented through the general health system. Pilot testing of practical approach to lung (PAL) strategy has been done in Kerala to improve the quality of diagnosis and treatment of common respiratory illnesses in primary health care (PHC) setting.[7] It seeks to standardize service delivery through development and implementation of clinical guidelines and managerial support within the general health system. In urban area RNTCP being implemented through involvement of medical colleges, private practitioners (intensified public private mix project being implemented in 14 states, mainly focusing referral of TB suspect, new smear positive case detection, DOT provision to TB patients and their treatment outcome).
- Human resource development by: (i) increasing the role of Medical Colleges in RNTCP program. A national task force, Zonal Task Force and State Task Force have been formed for training and oversee the functioning of the microscopy/treatment centre in their respective institutions, (ii) coordinating the TB-related and HIV/AIDS Training with the National AIDS Control Organization, (iii) training in advocacy, communication, and social mobilization: has crucial role in increasing the reach of services by involvement of other sectors, civil society organizations, NGOs, etc, creating conducive and patient friendly environment and also keeping the communities informed of the RNTCP services.
- Strengthening of health information system: (i) using already existing standard recording and reporting system, (ii) Training modules have been developed and regularly revised to train each and every health care provider under RNTCP, (iii) Managing information for action (MIFA): MIFA is an useful tool to equip District programme managers with the basic analytical and epidemiological skills necessary for processing and interpreting quality data and to effectively use those skills to manage their TB control program.[7] Further strengthening of health information system can be done by using electronic case-based recording and reporting systems using flexible computer solutions. These systems should be web based to allow remote data entry and real-time data management and reporting.
- Engaging all care providers: Activities that are on this road are (i) intensified public private mix (PPM) Project,[7] (ii) The Indian Medical Association (IMA) project is being implemented in 16 States, under the rolling continuation channel (RCC) project of global fund to fight AIDS, TB, and malaria (GFATM). IMA has endorsed the International Standards of TB Care and disseminated it widely in the country.[7] However, so far a very small section of the providers of TB care in the huge private health market of India have been involved and this remains as an important challenge for IMA and RNTCP. The IMA has also worked with other professional medical association and established the Indian Medical Professional Association Coalition against TB (IMPACT). (iii) RNTCP has signed a MOU with the Catholic Bishop’s Conference of India, Health Commission, for the ’First IMPACT’ TB Project under the guidance of the Central TB Division in 11 states.[7] (iv) Partnership for tuberculosis care and control: The partnership was set up in November 2008 and has a secretariat which provides administrative and technical support to the Partnership which currently includes 30 stakeholders across the country, representing NGOs, CBOs, FBOs, affected communities and private sector.[7] Experiences from other countries that could be useful in this direction are, in China where the mandatory case notification through an internet based communicable disease reporting system (IBCDRS) has doubled the case detection and also improved the follow up rates for referrals and suspect tracings.[8] In Ghana, Ghanaian food and drug board has put restriction on access to antitubercular drug in private sector through making sale of anti-tubercular drug non profitable and stopping antitubercular drug importation, resulted into decreased adverse treatment outcome and improved treatment success rate.[8]

#### Empowering people with TB, and communities through formulation and implementation in all states and districts ACSM (advocacy, communication and social mobilization) activities

Empowerment of the patient one of the weaker area. Some of the interventions which were assessed in other countries to empower the TB patients can be applied in Indian setting (i) giving information in culturally acceptable and in context of patient-centered approach, (ii) financial autonomy to TB patients and their family by disbursing small loans,[9] (iii) organizing TB patients into groups and clubs,[10] (iv) groups therapy sessions for multidrug-resistant TB patients,[11] (v) development of more patient-centered TB and general health care system by improving communication at patient and provider level, e.g., trialogue approach used in India.[12] (vi) enabling advocacy skill of TB patients to improve TB control by involving them in local planning, implementation and monitoring of the programme.

#### Enabling and promoting research into TB control activities

Major area of research for improving the TB surveillance system is to find out what fraction of cases are missed out from notification data. (i) To find out the number of cases that are being correctly diagnosed but not notified under RNTCP “inventory” studies can be conducted. These studies compare lists in which people suspected of having TB, TB cases, and TB deaths are recorded with lists of notified cases. Where such lists are not readily available other sources can be used such as hospital registers, HIV notification records with information on TB comorbidity, mycobacterial laboratory registers, prescriptions in pharmacies, information on drug sales in the private sector, health expenditure in the private or nongovernmental organization sectors, out-of-pocket expenditure, and proportion of health facilities not collaborating with the national TB control programme. If three or more lists can be generated capture–recapture methods may be used to estimate total incident cases. These methods found to be useful in estimation of not only the number of cases that are missing from notifications, but also the number of cases missing from all lists (i.e., cases that are not in contact with health facilities at all).[13–18] (ii) Survey should be conducted on knowledge, attitudes and practices of health staff in managing people suspected of having TB and cases that do not have access to health services. (iii) Although the prevalence survey for tuberculosis is difficult to conduct and it also does not provide the correct measures of incidence, but the periodic assessment through prevalence surveys give important information on short-term TB control measures. Whenever such surveys are conducted they can be used to establish the health-seeking behavior,[19] and extent to which identified cases already had contact with the health care system, the number that had not been diagnosed despite visiting health services and the number of cases that had not been notified due to health care providers not being linked to the national TB control programme. Such findings will help to identify the proportion of cases likely to be included in TB notification data, the reasons for lack of access to TB care and the absence of notification, and to develop interventions that will accelerate progress in TB control.

Considering the various limitations of Styblo’s rule and of other measures to estimate the incidence of sputum positive tuberculosis cases, development of an effective and complete surveillance system is the best tool for monitoring progress toward current tuberculosis control efforts. According to WHO report on Global Tuberculosis Control 2008,[20] India is on way of implementing all the four relevant components of Stop TB Strategy 2006, to develop a comprehensive and effective surveillance system, and this will be useful in future for more accurately measuring the epidemiological situation of Tuberculosis in India.
